# HIV p24 as Scaffold for Presenting Conformational HIV Env Antigens

**DOI:** 10.1371/journal.pone.0043318

**Published:** 2012-08-17

**Authors:** Maria Tagliamonte, Daniela Marasco, Alessia Ruggiero, Angelo De Stradis, Maria Lina Tornesello, Maxim Totrov, Franco Maria Buonaguro, Luigi Buonaguro

**Affiliations:** 1 Laboratory of Molecular Biology and Viral Oncogenesis, AIDS Reference Center, Istituto Nazionale Tumori “Fond. G. Pascale”, Naples, Italy; 2 Department of Biological Science-School of Biotechnological Sciences, University of Naples Federico II, Naples, Italy; 3 National Resource Council, Institute of Biostructure and Bioimaging, Naples, Italy; 4 National Resource Council, Institute of Plant Virology, Bari, Italy; 5 Molsoft LLC, La Jolla, California, United States of America; Commissariat a l’Energie Atomique(cea), France

## Abstract

Heterologous protein scaffolds engrafted with structurally defined HIV Env epitopes recognized by broadly neutralizing monoclonal antibodies (MAbs) represent a promising strategy to elicit broad neutralizing antibodies. In such regards, a protein scaffold based on the HIV p24 CA protein is a highly attractive approach, providing also Gag epitopes for eliciting HIV non-neutralizing protective antibodies and specific CD4^+^ and CD8^+^ T cell responses. In the present study, computational techniques were employed to verify the presence of acceptor sites for conformational HIV Env epitopes and, as proof of concept, the analysis of HIV p24 CA-based scaffolds using a complete V3 loop in a MAb-bound conformation is presented. The V3-p24 epitope-scaffold proteins show the formation of capsomers made of hexamers similarly to the p24 wild type protein. Moreover, the conformational V3 loop presented on p24 scaffold is recognized by a panel of anti-V3 MAbs. The results suggest that HIV p24 CA protein has suitable acceptor sites for engrafting foreign epitopes, without disrupting the formation of capsomer hexamer structures, and that the V3 epitope does retain its antibody-bound conformation. This strongly support the feasibility of developing a scaffolding strategy based on p24 CA proteins displaying conformational minimal structural, antigenic HIV Env epitopes.

## Introduction

Efforts to elicit protective immunity to HIV has resulted in unsatisfactory results [1]. In particular, elicitation of broadly reactive and cross-clade neutralizing antibodies (NAbs) is representing an unprecedented challenge for the intrinsic property of HIV to generate molecular and antigenic variants escaping the immune surveillance [2]. However, cross-reactive neutralizing antibodies targeting the envelope glycoprotein can indeed arise during the natural course of HIV-1 infection [3], [4], [5], [6], [7], as shown by the broadly neutralizing antibodies isolated from HIV-1-infected individuals. In particular, b12 and 2G12 bind to conserved epitopes in the gp120 subunit [8], [9]; 2F5 and 4E10 bind to conserved, contiguous epitopes in the gp41 subunit [10], [11]. More recently, additional broadly neutralizing antibodies have been described, targeting either discontinuous epitopes in trimeric structures (PG9 and PG16) [12], the CD4 binding site (HJ16, VRC01/2 and VRC03) [13], [14], or the V3 loop [15], [16], [17].

Strategies to elicit or expand such HIV broadly reactive and cross-clade NAbs are currently pursued by several groups, aiming at focusing the immune response on specific epitopes which can be either immunorecessive, cryptic or transiently exposed. To this goal, one of the optimal experimental strategies appears to be the selection of the minimal structural and antigenic epitopes, in order to isolate them from all other potential and confounding B-cell epitopes as well as from the shielding N-linked glycans within the whole HIV envelope glycoprotein [18], [19], [20], [21]. Such minimal epitopes, indeed, can be grafted in a constrained status onto appropriate heterologous protein scaffolds to mimic their antibody-bound conformation and be possibly able to elicit the counterpart broadly neutralizing Nabs.

Along such path, very recently the gp41 2F5-specific minimal epitope has been grafted on different protein scaffolds [22] inducing high titers of cross-reactive Ab response [23]. Similarly, the gp120 V3 loop has been grafted on a thioredoxin [24] or cholera toxin subunit (CTB) [25] scaffold, exhibiting high-affinity binding to a large panel of broad-neutralizing mAbs and inducing high titers of anti-V3 antibodies with broad-neutralization effect [25].

An additional relevant feature for a vaccine approach, aiming at an efficient induction of neutralizing antibodies, is to present B cell epitopes as dense, repetitive arrays mimicking the natural organization observed in viruses which induce highly protective neutralizing antibodies [26]. Densely repetitive B cell epitopes, indeed, induce also T cell-independent B cell activation in contrast to the same antigen presented in monomeric non-organized conformation, as shown in the Vescicular Stomatitis Virus (VSV) model [27].

In this perspective, Virus-Like Particles (VLPs) represent a highly attractive vaccine strategy, closely resembling authentic virions with a regular and rigid capsid structure presenting conformational viral epitopes as dense repetitive arrays [28], [29], [30], [31], [32]. However, antigen presentation on enveloped VLPs (i.e. HIV-VLPs) may be affected by a sparse and irregular distribution which reflects the structure of the authentic virions [33], [34], [35].

In order to overcome such limitation, non-enveloped particulate vaccines based on assembled chimeric HIV p24 Gag core protein can be prospected. Very recently, indeed, the hexameric structure of capsomers derived from in vitro assembling of recombinant HIV p24 capsid protein (p24 CA protein) has been described. HIV p24 CA proteins form homogenous populations of stable and soluble stand-alone capsomers, which assemble in vitro with the need of neither cellular membrane nor MA and NC gag viral proteins [36].

Based on such observations, the HIV p24 CA protein is a highly attractive molecule to be used as particulate protein scaffold for presenting dense repetitive arrays of minimal structural and antigenic HIV Env epitopes aiming at eliciting broadly NAbs [37]. Compared to other “neutral” structures, indeed, it would represent an invaluable advancement. In fact, besides the presentation of relevant Env neutralizing epitopes, it would provide also Gag epitopes for eliciting HIV non-neutralizing protective antibodies and specific CD4^+^ and CD8^+^ T cell responses. This would result in highly effective induction of both T cell-independent and T cell-dependent B cell activation, specific for both the grafted Env epitopes and the Gag-protein scaffold.

In this regards, the present paper describes the design and characterization of HIV p24-based scaffold displaying a full-length V3 loop epitope in the same conformation as bound to broadly NAbs.

## Materials and Methods

### Design of the Antigen Constructs

Conformation of the complete V3 loop in the gp120 context as revealed by the X-ray structure (PDB code 2B4C) [38] was used as a prototype to identify grafting site(s) on the scaffold. Coordinates of a pair of gp120 residues, N295 (immediately preceding the cysteine bridge C296–C331) and residue N332 (immediately following the bridge) were used to scan the HIV p24 CA structure (PDB code 3H47) [36] for a pair of residues closely matching the query in 3D configuration. In absence of such matching, a connector segment SSSDSG was designed to join V3 N-terminus with the N-terminal part of cyclophillin binding loop. Low RMSD matches were next subjected to a scaffold clash test: gp120, N295, and N332 were superimposed onto identified residue pairs in p24, and the resulting position of the V3 loop was checked for any clashes with the rest of the p24 scaffold. When low RMSD and clash-free superposition were achieved, the complete model of the chimeric protein was constructed in ICM using a regularization procedure that threads an idealized polypeptide chain through the template structures [39].

### Preparation of the Recombinant p24:V3 Constructs

The wild type (WT) HIV p24 CA as well as the chimeric p24:V3 proteins were synthesized and codon-optimized for expression in both prokaryotic and eukaryotic cells (GenScript Co., Piscataway, NJ USA). In particular, the chimeric p24:V3 proteins were mutated in the p24 sequence either at A14C/E45C (CC mutant) or at A14C/E45C/W184A/M185A (CCAA mutant), as previously described [36]. Synthetic WT and chimeric p24:V3 (CC and CCAA mutant) genes were cloned into pBiex plasmid (Novagen) and GST-tagged proteins were produced in E. coli strain BL21(DE3), transformed with each of recombinant plasmids. The IPTG induction was carried out at 22°C overnight, the cellular pellet was resuspended and lysed in a buffer containing 20 mM TrisHCl (pH 7.8), 200 mM NaCl, 0.01% (v/v) CHAPS, 5% (v/v) glycerol, and 1.0 mM âMe. GST-tagged proteins were purified by affinity chromatography on GST-trap column (5 ml, GeHealthcare) 4B. Before the GST-cleavage, the proteins were concentrated and loaded on a gel filtration column S200 (10∶30, GeHealthcare). Finally, tags were cleaved by an overnight enterokinase digestion. The protein mixture was further purified on affinity chromatography where cleaved proteins were collected in the flow-through and stored at −80°.

**Figure 1:**
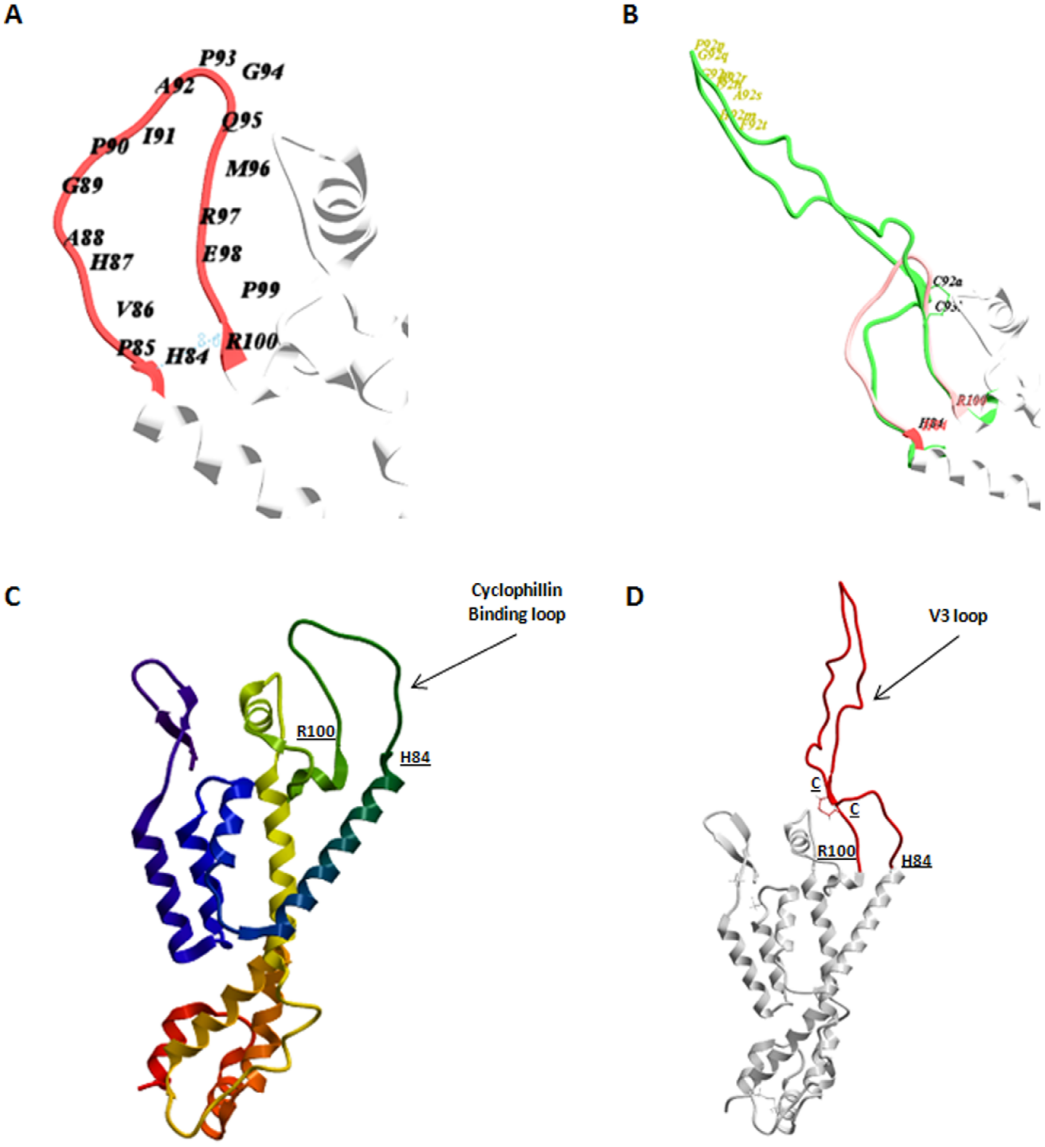
Identification of acceptor site in the p24 CA protein. The cyclophillin binding loop on the p24 CA protein is identified between the boundary residues H84 and R100 (PDB accession code 3GV2) (**A**). The full V3 loop sequence (green) as observed in the gp120 X-ray structure (PDB accession code 2B4C) has been introduced between the H84 and R100 residues and superimposed to the cyclophillin binding loop (red). Side view (**B**). The cysteins at the base of the V3 loop as well as the sequence at the tip of the V3 loop are indicated. The overall wild type (**C**) and chimeric (**D**) p24 structures are shown.

### Characterization of Double-Cysteine Mutants

Proteins were assembled in vitro either by direct dilution [40] or by overnight dialysis [41] into assembly buffer (50 mM Tris [pH 8], 1 M NaCl) containing 20–200 mM beta ME. Assembled particles were visualized by transmission EM, as previously described [42]. Alternatively, crosslinking of proteins was achieved by subsequent dialysis into assembly buffer with the beta ME concentration reduced to 20 mM or lower. The extent and efficiency of crosslinking was assessed by non-reducing SDS-PAGE, mixing the samples in running buffer with 20 mM beta ME. Gag p24 and Env V3 loop expression were evaluated by WB analysis incubating blotted proteins with a 1∶400 dilution of rabbit polyclonal anti-p24 anti-serum (ARP432, NIBSC Centralised Facility for AIDS Reagents, Hertfordshire, UK) or human monoclonal anti-V3 antibodies (ARP3023, ARP3024 and ARP3119, NIBSC Centralised Facility for AIDS Reagents, Hertfordshire, UK). After an overnight incubation, secondary peroxidase-conjugated goat anti-rabbit and anti-human IgG, respectively (Santa Cruz Biotechnology Inc., Santa Cruz, CA) (1∶1000 dilution) were added for 2 hours. After extensive washes with 1×TBS, bound antibodies were visualized adding 4-chloro-1-naphtol as substrate.

**Figure 2:**
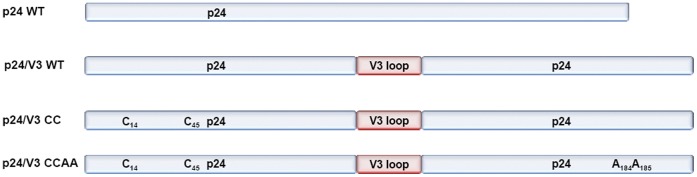
Amino acid sequence of the p24:V3 chimeras. The amino acid sequence of wild type and mutant p24:V3 chimera are indicated. Mutated residues in CC and CCAA mutants are underlined. The V3 loop sequence inserted in the p24 sequence is indicated in the box.

Gel filtration analyses were carried out loading proteins onto a Superdex-200 PC column (GeHealthcare) equilibrated in a buffer with 30 mM Tris (pH 8.0), 300 mM NaCl, and 2 mM bME at flow rate of 0.04 mL/min.

### Electron Microscopy

For negatively stained TEM images, the assembled particles were adsorbed to Formvar carbon-coated copper grids by floating the grids on a drop of each sample for 30 s. The grids were rinsed with three drops of 0.1 M KCl, touched to Whatman filter paper, rinsed with three drops of saturated uranyl acetate and dried on Whatman filter paper. Observations were performed with FEI Morgagni 282D electron microscope operating at 60 Kv.

**Figure 3:**
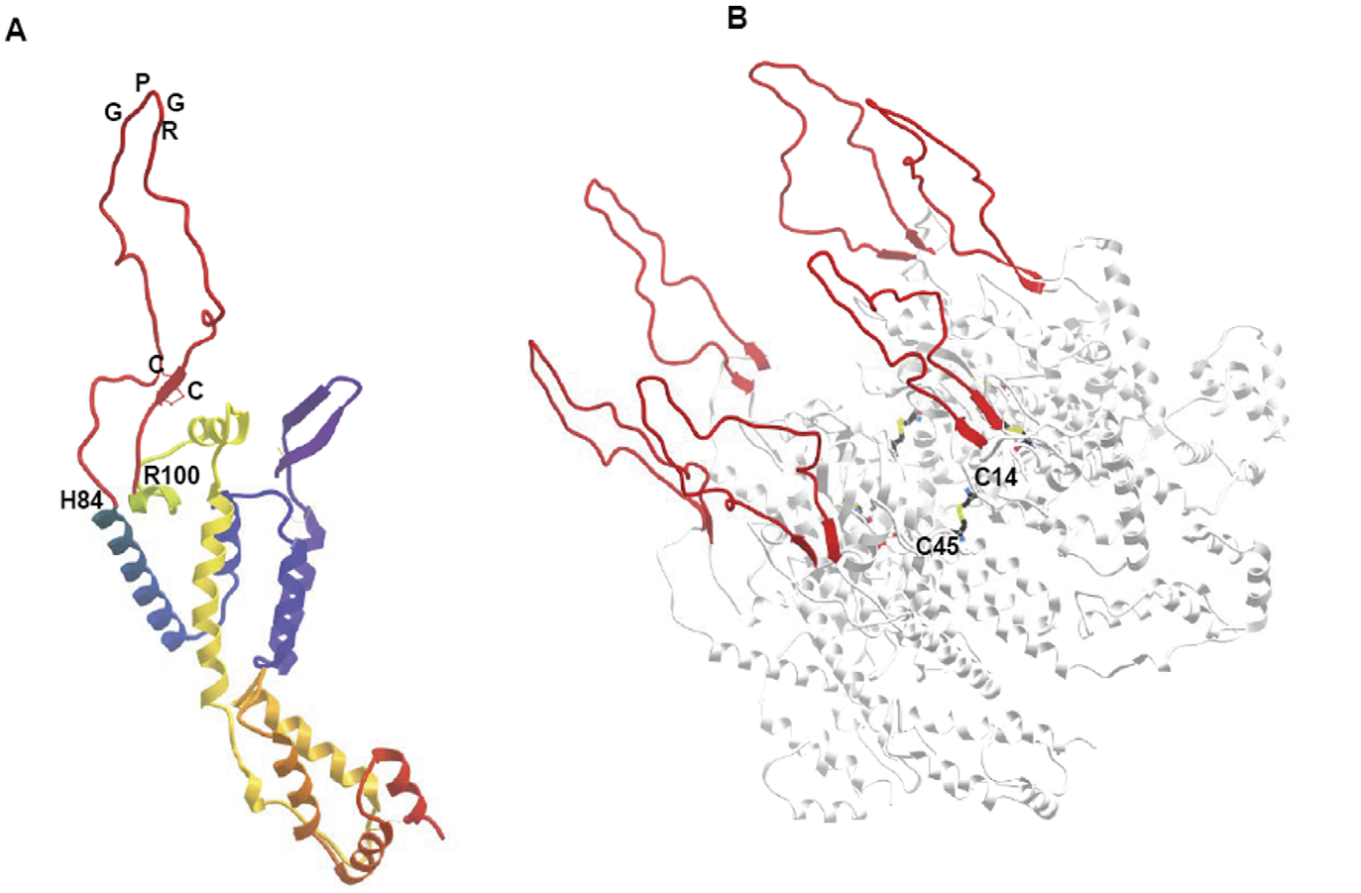
Display of the V3 loop by the p24 scaffold. The conformation of the V3 loop sequence inserted in the p24 CA protein within the residues H84 and R100 is shown as monomer (**A**) and as hexameric structure (**B**). The mutated cysteins in position 14 and 45 of two adjacent p24 CA monomers are indicated in the hexamer (**B**).

In parallel, assembled particles were prepared for ultrathin sectioning according to standard procedures [43]. In particular, cells were fixed in ice-bath for 1 h in 2.5% glutaraldehyde in 0.05 M phosphate buffer, post-fixed in 1% osmium tetroxide, stained overnight in 0.5% aqueous uranyl acetate, dehydrated in graded ethanol dilutions and embedded in TAAB SPURR RESIN with ERL 4221D-medium grade (Agar Scientific, UK). Thin sections were stained with lead citrate prior to viewing under the FEI MORGAGNI 282D electron microscope using an accelerating voltage of 60 kV.

**Figure 4:**
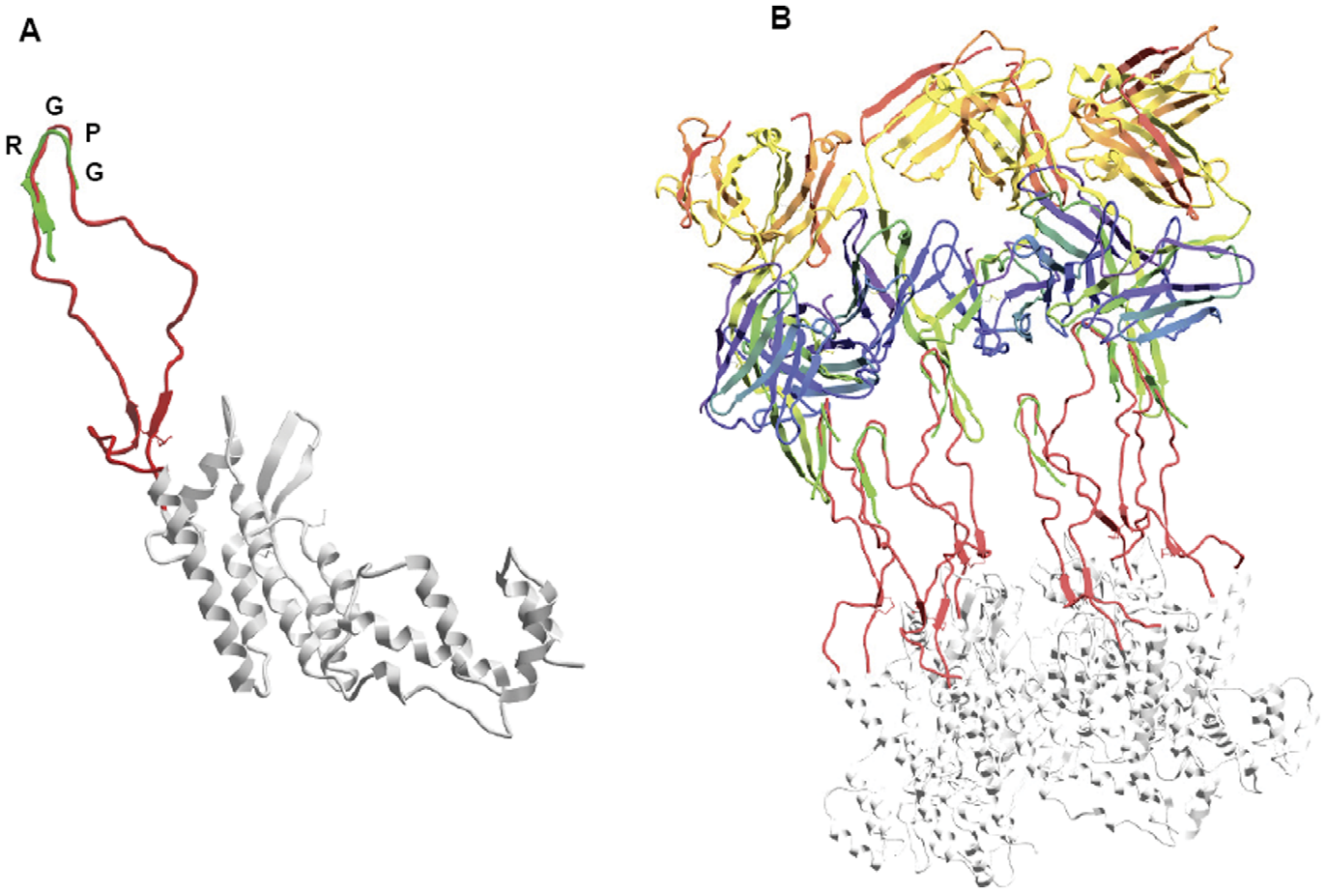
The engrafted V3 loop sequence is fitting to the mAb 447-52D. The engrafted V3 loop sequence (in red) is superimposed to the V3 crystallized bound to the Mab 447-52d (PDB 1Q1J) (in green) (**A**). The six engrafted V3 loop in the hexameric structure can be bound by independent mAbs. For clarity, only three out of six are indicated (**B**).

### Evaluation of Binding of p24:V3 Proteins to Anti-V3 MAbs

#### ELISA

Chimeric p24:V3 (CC and CCAA mutant) and wild type p24 proteins were coated onto plates at 0.1 µg/ml and incubated overnight at 4°C. After three cycles with wash buffer (1×PBS with 0.05% Tween 20, pH 7.4), nonspecific protein binding to the plates was blocked by incubation with 5% (wt/vol) dry milk in PBS for 1 h at room temperature (RT). Plates were incubated for 3 h at 37°C with human anti-V3 mAbs at concentrations ranging from 10 pg to 10 ng/ml. The anti-V3 mAbs are 257-D IV (ARP3023) which recognizes the KRIHI sequence, 268-D IV (ARP3024) which recognizes the HIGPGR sequence and 447-52D (ARP3219) which recognizes the GPGR sequence. After washing, plates were incubated with alkaline phosphatase-conjugated goat anti-human IgG (Fc-specific) for additional 1.5 h at 37°C. After three washings, reactions were visualized by adding the substrate p-nitrophenyl phosphate in 10% diethanolamine for 30 min and plates were read at 410 nm. Negative controls consisted of wells coated with two distinct p24 proteins reacted with the three anti-V3 mAbs at the same concentrations as for p24:V3 chimera. Cut-off values were considered as 2-fold the optical density observed on the p24 proteins. Data points were in triplicates or duplicates and reported experiments are from three independent assays.

**Figure 5:**
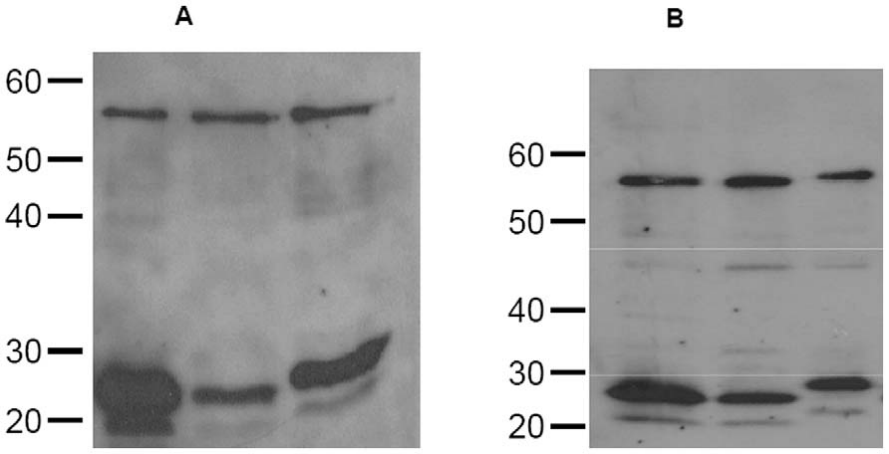
Expression of p24:V3 chimera in E.coli. GST-tagged proteins were purified on GST trap and tags were cleaved by enterokinase digestion. Cleaved p24:V3 protein mixtures were tested on 10% SDS PAGE and western blot analysis was performed using a mix of the human anti-V3 mAbs (**A**) or rabbit polyclonal anti-p24 antibody (**B**). The higher band represents the uncleaved GST-protein fraction.

#### SPR analysis

The BIAcore 3000 SPR system for Real time binding assay and related reagents were from GE Healthcare (Milano, Italy). Anti-V3 mAb 447-52D (ARP3219) was immobilized at a concentration of 20 µg/ml in 10 mM acetate buffer pH 5.5 (flow rate 5 ul/min, time injection 7 min) on a CM5 Biacore sensor chip, using EDC/NHS chemistry following the manufacturer’s instructions [44]. Residual reactive groups were deactivated by treatment with 1 M ethanolamine hydrochloride, pH 8.5. Reference channel was prepared by activating with EDC/NHS and deactivating with ethanolamine. Binding assays were carried out at 20 µl/min, with 4,5 min contact-time, p24 V3 chimeric proteins were diluted in the running buffer, HBS (10 mM Hepes, 150 mM NaCl, 3 mM EDTA, pH 7.4), 0.1 mM TCEP. Analyte injections of 90 ul were performed at the indicated concentrations. The sensor surface was regenerated by using 1–3 washes of 10 mM NaOH for 1 minute. BIAevaluation analysis package (version 4.1, GE Healthcare, Milano, Italy) implemented by instrument software was used to subtract the signal of the reference channel and to evaluate kinetic parameters (kon and koff) from which we derived the values of thermodynamic dissociation constants of complexes, by fitting the data to a 1∶1 Langmuir interaction model.

**Figure 6:**
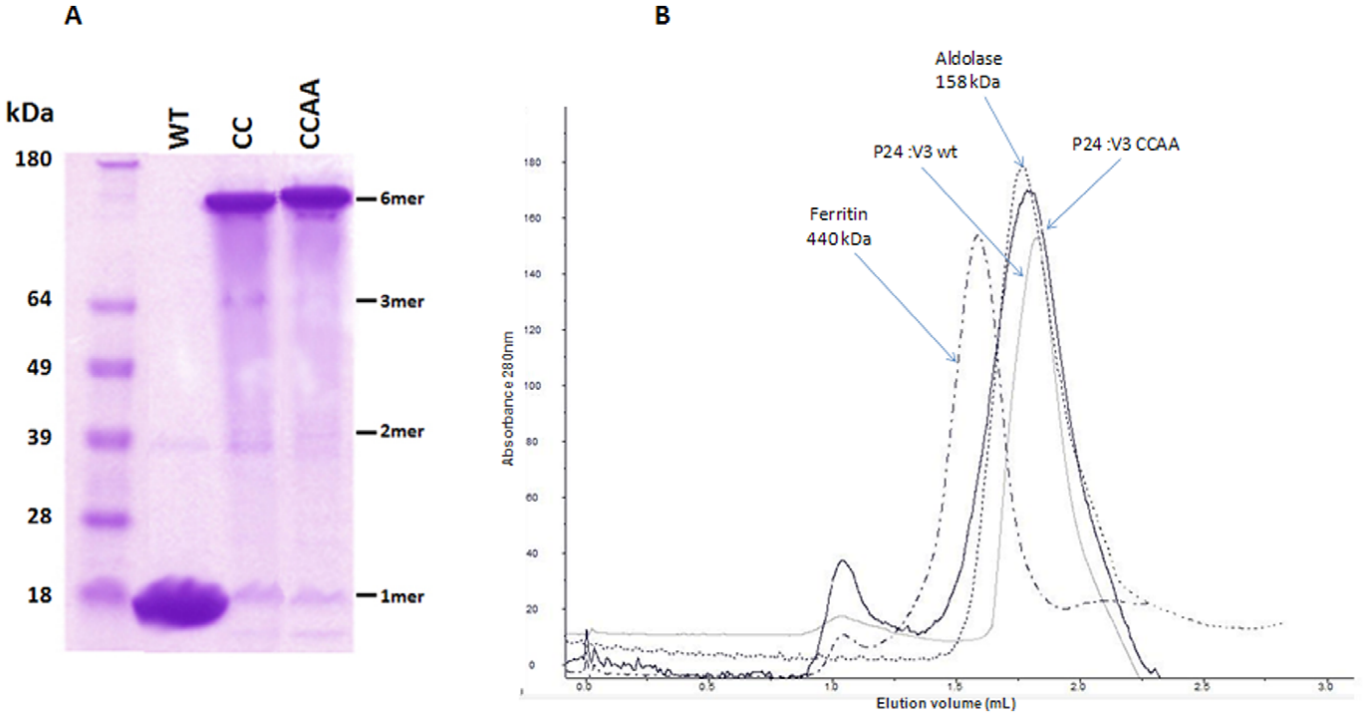
(**A**) Crosslinking of mutant p24:V3 chimera. Proteins were separated in non reducing SDS-PAGE conditions on a 10% polyacrilamide gel. The p24:V3 CC and CCAA mutants migrate almost exclusively as hexamers, while the p24:V3 WT chimera migrates as monomer. (**B**) Size exclusion chromatographic profile. Profile of p24:V3 WT (gray) and p24:V3 CCAA mutant (black) on Superdex-200 PC column is shown. Elution volumes of protein standards such as ferritin (440 kDa,dashed and dotted line) and aldolase (158 kDa, dotted line) are also indicated.

## Results

### Identification of an Acceptor Site in the HIV p24 Protein

The polypeptide backbone in the X-ray structure of the HIV p24 Capsid protein (PDB accession code 3GV2) [36] has been scanned for an acceptor site suitable to engraft the V3 loop in a conformational status. The candidate site has been identified in the cyclophillin binding loop (I73–S110) which is located on the surface of the HIV capsid, on the outer edge of the p24 CA hexamer [45] (Fig. 1A). In particular, the residues H84 and R100 appear the optimal structural boundaries for inserting the full V3 loop sequence to residue positions that would allow low RMSD superposition and clash-free match with the termini of the V3 loop as observed in the gp120 X-ray structure (PDB accession code 2B4C) [38] (Fig. 1B). Moreover, the substitution of the cyclophillin binding loop with the V3 loop is predicted not inducing any conformational change in the overall p24 structure (Fig. 1C and 1D).

**Figure 7:**
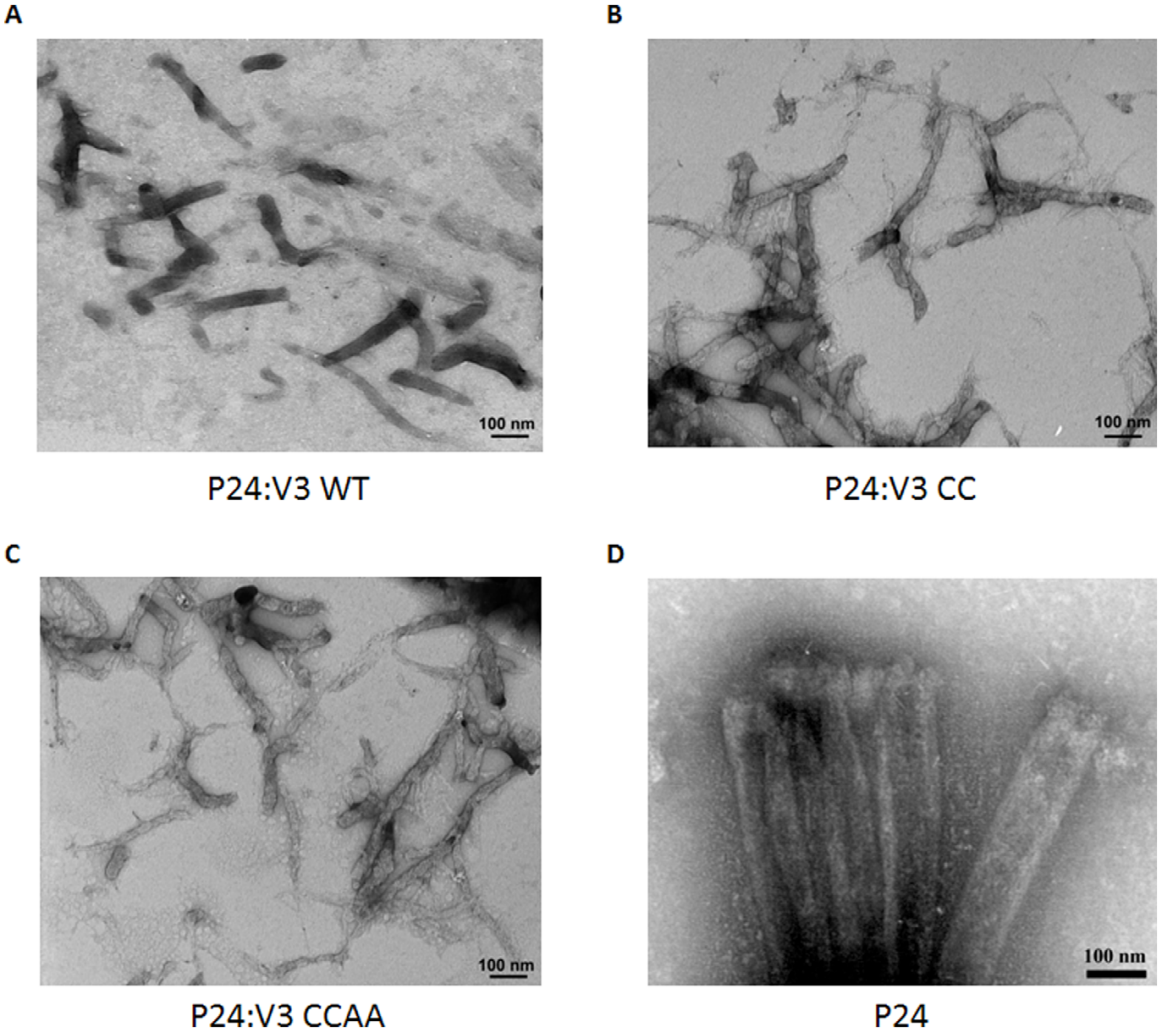
Electron microscope analysis. P24:V3 chimeric proteins assembled in vitro were stained with uranyl acetate, and visualized via transmission electron microscopy. They form cylinder structures similar to the ones formed by the p24. The scale bars are indicated.

### Design of Chimeric HIV p24:V3 Proteins

According to such prediction analysis, a chimeric sequence was designed based on the HIV p24 CA protein where the full V3 loop with V3 terminal cysteines C296 and C331 was introduced to replace the cyclophillin binding loop between the p24 residues H84 and R100 (Fig. 2). In addition to the wild-type p24 sequence, two chimera sequences have designed in which the p24 sequence has been mutated in A14C/E45C (CC mutant) or in A14C/E45C/W184A/M185A (CCAA mutant). Such mutations, indeed, have been previously shown to stabilize the hexameric structure formed by the p24 CA protein(CC mutant) and to weaken the inter-hexameric structure while leaving the hexamer-stabilizing interfaces unchanged further (CCAA mutant) [36].

As predicted, the chimeric p24:V3 protein displays the V3 loop in a conformation as observed in the gp120 X-ray structure (Fig. 3A) and the reconstruction of the complete hexamer, by superimposing six subunits of the chimeras, showed that the engrafted loop did not interfere with the assembly (Fig. 3B). Moreover, the six V3 loops projected from the hexameric scaffold without any interaction or conformational hindrance and the A14C/E45C mutations are confirmed to form inter-subunit disulfide bonds (Fig. 3B).

**Figure 8:**
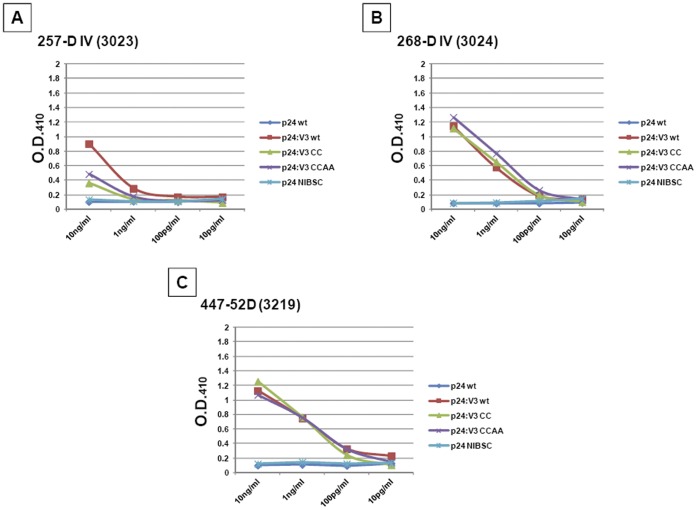
Reactivity of the p24:V3 chimera with anti-V3 mAbs. Plates were coated with 10 ng of each protein and mAbs were added to each well at indicated titrated concentrations. Cut-off values were considered as 2-fold the optical density observed on the negative controls (p24 proteins).

### The Tip of Engrafted V3 Loop is Conformationally Fitting with the mAb 447-52D

The engrafted full V3 loop has been subsequently superimposed to the V3 loop fragment K305-A316 found to bind to the Fab fragment of the broadly NAb 447-52D in the X-ray structure (PDB accession code 1Q1J) [21], [46]. The two V3 structures, indeed, show a very significant superimposition as observed considering the p24:V3 chimera as single molecule (Fig. 4A). Moreover, the hexameric structure formed by the assembled p24:V3 molecules showed that the spatial conformation allows the 447-52D mAb to interact simultaneously and independently with each of the six engrafted V3 loops (Fig. 4B).

Such observation strongly suggest that the p24 is a suitable scaffold and its hexamers efficiently display an array of V3 antigens for optimal induction of T-cell dependent as well as independent B cell response and generation of antibodies with potential broad neutralising activity.

### Analysis of the Expression of p24:V3 Chimeric Proteins

The genes for the three p24:V3 chimeric proteins have been synthesized and codon-optimized for expression in both prokaryotic and eukaryotic cells. After cloning into pBiex plasmid GST-tagged proteins were produced in *E.coli* strain BL21(DE3) and purified by affinity chromatography on GST trap. Tags were cleaved by enterokinase and digested samples were analyzed on SDS-PAGE in both reducing and non-reducing conditions.

The WB results, obtained on protein samples analyzed in reducing conditions, show the expression of the three proteins with the expected molecular weight, which are recognized by both the anti-V3 447-52D mAb (Fig. 5A) and the anti-Gag polyclonal Antibody (ARP432) (Fig. 5B).

Furthermore, the results of SDS-PAGE analysis performed in non-reducing conditions show that the amino acid substitutions introduced in the p24:V3 CC and CCAA mutants induce a strong cross-linking and molecules migrate almost exclusively as hexamers. In the same experimental conditions, the hexamers of p24:V3 WT chimera are dissociated and migrate as monomers (Fig. 6A). On the contrary, p24:V3 WT and CCAA constructs, when analyzed in solution in a gel filtration analysis, show an elution pattern comparable to aldolase (158 kDa), suggesting that in such experimental conditions they are in a hexameric status (Fig. 6B). Similar pattern is observed with the CC mutant (data not shown).

### Characterization of p24:V3 Chimeric Proteins by Electron Microscopy Analysis

The self-assembly state of p24:V3 chimeric proteins was investigated by electron microscopy analysis. The CC mutant assembled into cylinders that were similar to those formed by the p24:V3 WT protein (Fig. 7A). Also the two mutants assembled into long hollow cylinders (Fig. 7B and 7C), showing that the mutations introduced in the p24 sequence do not alter the assembly of the molecule into helical arrays of hexamers (Fig. 7D) [36], [47].

### Binding of the p24:V3 Chimeric Proteins with Anti-V3 mAbs by ELISA and SPR

ELISA experiments have been performed to evaluate the reactivity of different anti-V3 mAbs to the three p24:V3 chimera and results are presented in Fig 8.

Three different MAbs specific for the V3 loop sequence have been used in a titration from 10 pg to 10 ng/ml and the results show a clear binding by all three MAbs to the three p24:V3 chimera proteins coated at a concentration of 0.1 µg/ml (final 10 ng), suggesting that the V3 loop inserted on the p24 scaffold is was sufficiently exposed and retained the ability to bind to anti-V3 mAbs. As expected, the 447-52D (3219) MAb, which is the reference structure used to build the present V3-p24 epitope-scaffold, shows the highest values against all three p24:V3 chimeras. The 268-D IV (3024) MAb, which share with the 447-52D (3219) MAb the binding V3 GPGR epitope, shows similar OD values against all three p24:V3 chimeras. On the contrary, the 257-D IV (ARP3023) MAb, which binds to the adjacent V3 KRIHI epitope, shows lower OD values especially against the the p24:V3 CC and CCAA mutants (Fig. 8).

**Figure 9:**
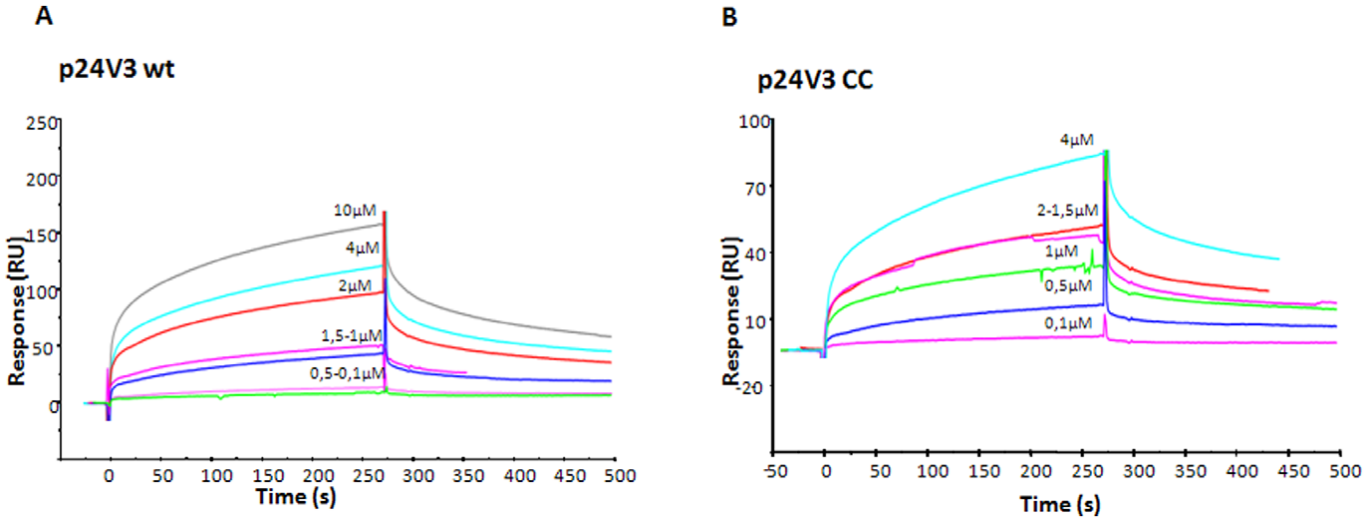
SPR analysis of anti-V3 447-52DmAb with p24:V3 chimera. Overlay of sensorgrams relative to the direct binding of p24:V3 wt (**A**) and p24:V3 CC (**B**) to immobilized antiV3 447-52D (3219) mAb. Concentrations were reported on the sensorgrams. Experiments were carried out in duplicate, at a 25°C, at a constant flow rate of 20 uL/min using HBS as running buffer.

In order to confirm ELISA binding and to quantify the entity of the binding between antiV3 447-52D (3219) mAb and p24:V3 chimeric proteins, SPR experiments were carried out (Fig. 9). The mAb was efficiently immobilized on CM5 (averaged immobilization level 2000 RU) and p24:V3 wt and p24:V3 CC were employed as analyte (concentration range 0.10–4.0 µM). Dose-response sensorgrams revealed that both chimeric proteins bound anti V3 mAb in a quite similar fashion providing a K_D_ values of 10.05±0.8 and 13.40±0.3 µM for p24:V3 wt and p24:V3 CC, respectively (Fig. 9 A and 9B), confirming that V3 epitope is well exposed in chimeric proteins. The p24:V3 CCAA mutant showed K_D_ values similar to those of p24:V3 CC mutant (data not shown).

## Discussion

The engraftment of HIV envelope epitopes onto heterologous protein scaffolds in a suitable conformation to recognize broadly neutralizing antibodies, as identified by crystallographic analysis, has been reported by several groups [48], [21], [18], [23], [25], [22].

Here we describe the design and characterization of a novel scaffold based on the HIV p24 CA protein. This approach, besides the presentation of relevant Env neutralizing epitopes, would provide also Gag epitopes for eliciting HIV non-neutralizing protective antibodies and specific CD4+ and CD8+ T cell responses. The ultimate result would be the effective induction of both T cell-independent and T cell-dependent B cell activation, specific for both the grafted Env epitopes and the Gag-protein scaffold. Moreover, HIV p24 CA proteins have been previously shown to assemble into non-enveloped capsomers made of hexameric structures [36] which may present dense repetitive arrays of minimal structural and antigenic HIV env epitopes aiming at eliciting broadly NAbs.

In the present study, the HIV V3 loop has been selected as antigenic epitope to be engrafted on the p24 CA protein. The suitable acceptor site on the p24 CA protein has been predicted by bionformatic modeling in the cyclophillin binding loop located on the surface of the HIV capsid, on the outer edge of the p24 CA hexamer. Bioinformatic analyses, indeed, predict that the V3 loop peptide inserted between the residues H84 and R100, representing the boundaries of cyclophillin binding loop, is conformationally superimposed to the V3 loop as observed in the gp120 X-ray structure. Moreover, the tip of the engrafted V3 loop would be conformationally superimposed to the V3 loop fragment K305-A316 bound to the Fab fragment of the broadly NAb 447-52D in the X-ray structure. Such results, indeed, show that the p24 CA protein would have an acceptor site suitable for engrafting HIV envelope epitopes, retaining the same conformation as in the native whole gp120 molecule as well as in the bound status to broadly neutralizing Abs.

The substitution of the cyclophillin binding loop with the V3 loop would not interfere with the p24 assembly into hexameric capsomers and the six V3 loops displayed by p24 CA proteins in each hexamer would project from the scaffold without any interaction or conformational hindrance. Moreover, the spatial distribution predicts that six 447-52D mAbs would interact simultaneously and independently with the six engrafted V3 loops.

Such bioinformatic analyses suggest that the HIV p24 CA could be exploited as effective scaffold for presenting arrays of minimal structural and antigenic HIV Env B cell epitopes aiming at eliciting broadly NAbs, as described for native viruses [26]. This modality of presentation, indeed, has been shown to induce B cell activation and antibody production not only in T cell-dependent but also in T cell-independent fashion, by which B cells mount strong and rapid responses to multivalent antigens without the help of CD4+ T cells (reviewed in [49], [50]). Moreover, in the absence of infection or intracellular replication, structured antigens such as capsomers or Virus-Like Particles are able to enter both the extracellular and the endogenous processing pathway within antigen presenting cells (APCs), activating also antigen-specific CD8+ cytotoxic T lymphocytes (CTL), a phenomenon known as cross-priming [51], [52]. It is therefore expected that scaffolds based on p24 CA proteins would be able to efficiently induce not only Abs against the displayed conformational envelope epitopes but also cellular responses against both envelope and p24 CA epitopes.

Chimeric p24:V3 proteins have been expressed also in mutated forms substituting A14C/E45C (CC mutant) or A14C/E45C/W184A/M185A (CCAA mutant), which have been previously reported to stabilize the hexameric structure formed by the p24 CA protein(CC mutant) and to weaken the inter-hexameric structure while leaving the hexamer-stabilizing interfaces unchanged (CCAA mutant) [36]. Our results confirm that introduction of two cystein residues in position 14 and 45 of the p24 CA generates proteins able to cross-link in non-reducing conditions through the formation of disulphide bonds. On the contrary, the wt and mutant chimeric p24:V3 proteins show a comparable hexameric status in solution, suggesting that the three molecules should retain the capsomer structure at conditions of antigen administration in vivo. Moreover, these mutant proteins display the engrafted V3 loop epitope with similar characteristics, as shown by binding efficacy to different mAbs in ELISA. Moreover, similar binding affinity values of p24:V3 chimeric proteins to antiV3 447-52D (3219) mAb, further suggest that the conformation of V3 in the three molecules is, indeed, comparable.

These results serves as an important proof-of-concept for immunogen design based on the HIV p24 scaffold for eliciting optimal immune response against key conformational epitopes recognized by naturally occurring broadly neutralizing antibodies. Moreover, a variety of HIV envelope sequences and structures are planned to be engrafted on such p24 scaffold to evaluate the breadth and potency of the elicited Ab response in vivo. In conclusion, the overall data suggest that this scaffolding approach is potentially very promising as HIV vaccine strategy.
